# Histone H3K9 Trimethylation Downregulates the Expression of Brain-Derived Neurotrophic Factor in the Dorsal Hippocampus and Impairs Memory Formation During Anaesthesia and Surgery

**DOI:** 10.3389/fnmol.2019.00246

**Published:** 2019-10-25

**Authors:** Tong Wu, Xiao-Yu Sun, Xiu Yang, Le Liu, Kun Tong, Ya Gao, Jing-Ru Hao, Jing Cao, Can Gao

**Affiliations:** ^1^Jiangsu Province Key Laboratory of Anesthesiology, Jiangsu Province Key Laboratory of Anesthesia and Analgesia Application, Xuzhou Medical University, Xuzhou, China; ^2^Department of Anesthesia, The Affiliated Hospital of Xuzhou Medical University, Xuzhou, China; ^3^Department of Anesthesia, Xuzhou Central Hospital, The Xuzhou Clinical College of Xuzhou Medical University, Xuzhou, China

**Keywords:** anaesthesia and surgery, H3K9, trimethylation, brain-derived neurotrophic factor, hippocampus, memory acquisition

## Abstract

Brain-derived neurotrophic factor (BDNF) is essential for cognitive and memory functions. Abnormal BDNF expression in the central nervous system may impair these functions. Anaesthesia and surgery can induce perioperative neurocognitive disorders (PND). Clinical studies show that BDNF expression is decreased in patients presenting with cognitive impairment after anaesthesia and surgery. However, the molecular mechanism is still unclear. Epigenetic regulation plays an important role in cognition. The hypermethylation of H3K9 is crucial for transcriptional silencing and the onset of cognitive disorders. Here, we hypothesised that H3K9 trimethylation repressed BDNF expression and impaired memory formation or recall during anaesthesia and surgery. Laparotomy under isoflurane inhalation anaesthesia, behavioural tests, Western blotting, quantitative real-time reverse-transcription polymerase chain reaction (qRT-PCR), chromatin immunoprecipitation (ChIP), and immunohistochemistry were used in this study. BDNF expression was decreased in the hippocampus after anaesthesia and surgery. Cognitive impairment affected memory formation but not recall. The trimethylation of H3K9 downregulated BDNF expression. The overexpression of BDNF or use of exogenous BDNF improved the impairment of memory formation caused by anaesthesia and surgery. Therefore, inhibiting H3K9 trimethylation and increasing the expression of BDNF may help prevent PND in the clinical setting.

## Introduction

Postoperative cognitive changes are a common complication, especially in elderly patients who undergo major surgeries, including arthroplasty, laparotomy, and thoracotomy with or without cardiopulmonary bypass (Saczynski et al., 2012; Evered et al., 2018). Perioperative neurocognitive disorders (PND) are characterised by symptoms such as disturbance of memory, attention, consciousness, information processing, and the sleep-wake cycle, leading to postoperative morbidity and mortality (Evered et al., 2018). PND occurs predominantly in elderly patients but may also occur in other age groups (Johnson et al., 2002; Backeljauw et al., 2015). Multiple factors may be involved in PND (Cibelli et al., 2010; Han et al., 2014; Li et al., 2015); however, the molecular mechanisms underlying this condition are unclear.

Under some circumstances, general anaesthetics may cause long-term cognitive impairment (Li et al., 2014; Zhong et al., 2014; Cao et al., 2018) but have also been shown to promote brain protection (Fukuda and Warner, 2007), indicating that anaesthetics may play different roles in PND.

Cognitive processes are complex. In humans, these processes can be divided into five dimensions: attention, perception, memory, language, and learning. In clinical research, memory impairment after anaesthesia and surgery is a typical symptom and is often used as the main diagnostic criterion for PND (Evered et al., 2018). Memory functions involve formation, consolidation, storage, and recall. Brain-derived neurotrophic factor (BDNF) is implicated in several adaptive and pathological processes (Harward et al., 2016; Tanqueiro et al., 2018; Lima Giacobbo et al., 2019; Oh et al., 2019). An increasing number of studies have demonstrated that BDNF in the hippocampus, especially in the dorsal CA1 region, plays a key role in regulating cognition and memory (Bambah-Mukku et al., 2014; Bekinschtein et al., 2014; Reimers et al., 2014). A clinical study reported that BDNF protein expression decreased after anaesthesia and surgery in patients with cognitive impairment (Wyrobek et al., 2017).

Multiple amino acid residues on histone H3 can be modified in cognitive processes (Hyun et al., 2017; Kim and Kaang, 2017). Lysine 9 in H3 (H3K9) can be acetylated or methylated (Kilpinen et al., 2013; Lanouette et al., 2014). Theoretically, the deacetylation or hypermethylation of H3K9 can induce chromatin condensation, resulting in long-term gene silencing, and the trimethylation of H3K9 (H3K9me3) mediates transcriptional silencing (Karmodiya et al., 2012; Zovkic and Sweatt, 2013; Zovkic et al., 2013). In contrast, the acetylation and demethylation of H3K9 activate transcription (Ushijima et al., 2012). SUV39H is a typical histone methyltransferase involved in H3K9 trimethylation and promotes gene silencing. Moreover, H3K9 trimethylation is critical for cognitive impairment and is involved in the transcriptional repression of the *Bdnf* gene (Kuzumaki et al., 2011; Gupta-Agarwal et al., 2012; Maddox et al., 2013; Karpova, 2014). Considering that H3K9 is located near the promotors, modifications in this histone affect DNA methylation and transcription (Du et al., 2015; Zhao et al., 2016).

This study assessed whether the trimethylation of H3K9 was involved in the downregulation of BDNF expression leading to cognitive and memory impairment, and the stage at which memory processing was affected by anaesthesia and surgery. H3K9 trimethylation downregulated BDNF expression and impaired memory formation, but not recall, during anaesthesia and surgery. Therefore, inhibiting H3K9 trimethylation and increasing the expression of BDNF may help prevent PND in a clinical setting.

## Materials and Methods

### Animals

Adult male C57BL/6J mice (10–12 months old) and adult male vGLUT1-IRES-CreERT mice (homozygous, C57BL/6J background, 10–12 months old) were obtained from Xuzhou Medical University Animal Center (Xuzhou, China). All mice (five animals per cage) were acclimatised under a 12–12 h light-dark cycle and were allowed *ad libitum* access to food and water. All behavioural experiments were carried out between 8:00 am and 5:00 pm. All protocols were approved by the Animal Welfare Committee of Xuzhou Medical University and complied with Animal Research: Reporting of *in vivo* Experiments guidelines.

### Drugs and Primers

Chaetocin (Sigma-Aldrich, USA; 3 μg in 0.3 μl of 10% DMSO in PBS) was microinjected into the dorsal CA1 region of the hippocampus on each side 2 h before the tests. Recombinant BDNF (R&D Systems, USA; 0.5 μg in 0.3 μl of distilled water) was microinjected into the dorsal CA1 region of the hippocampus on each side 1 h before the tests.

The following primers were used in RT-PCR: total BDNF, 5′-AAGGACGCGGACTTGTACAC-3′ (forward), 5′-CGCTAATACTGTCACACACGC-3′ (reverse); GAPDH, 5′-AGGTCGGTGTGAACGGATTTG-3′ (forward), 5’-TGTAGACCATGTAGTTGAGGTCA-3′ (reverse); The primers for chromatin immunoprecipitation (ChIP) IV, 5′-AAAAACGGTCCAAAGACCAC-3′ (forward), 5′-TCACTAAGCCCCCTTCCTCT-3′ (reverse).

### Anaesthesia and Surgery

Animals were placed on a transparent chamber prefilled with 1.4% isoflurane and 100% oxygen (Peng et al., 2016). After a 15-min induction, a simple laparotomy was performed. A 0.5-cm longitudinal midline incision was made from the xiphoid to the proximal pubic symphysis on the skin, abdominal muscles, and peritoneum. Then, the incision was sutured layer by layer. Compound lidocaine cream (2.5% lidocaine and 2.5% prilocaine) was applied to the incision wound every 8 h for 2 days. The surgical procedure lasted approximately 20 min for each mouse, and the animals remained under anaesthesia for up to 2 h. The temperature of the anaesthesia chamber was controlled to maintain the rectal temperature of the mice at 37 ± 0.5°C during anaesthesia/surgery. The concentrations of isoflurane and oxygen and animal breathing were monitored. The animals from the control group remained in a chamber filled with 1.4% isoflurane and 100% oxygen for 2 h but were not subjected to surgery.

### Open Field Test

An open field test (OFT) was used to determine the spontaneous locomotor activity of mice according to a previous study (Wang et al., 2019). The OFT apparatus consisted of a 50 × 50 cm open arena with 50-cm-high walls, and the floor was divided into nine squares of equal areas. Briefly, the mice were individually placed into the centre of the apparatus and were free to explore the environment for 10 min. Thereafter, the total distance travelled was recorded by ANY-maze software during the following 5-min period.

### Y-Maze Test

The maze consisted of three arms (8 × 30 × 15 cm, width × length × height) that were 120° from each other: the start arm (always open), novel arm (closed during training and open during tests), and the other arm (always open). During training, mice were allowed to explore the maze for 10 min. After a 2-h interval, the animals were returned to the start arm for a 5-min exploration with the novel arm open. The number of entries and time spent on each arm were recorded.

### Novel Object Recognition Test

A novel object recognition (NOR) test was performed using a 50 × 50 cm open arena with 50-cm-high walls. The arena was divided into four quadrants, and two identical objects were placed diagonally from each other. Mice were individually placed into the centre of the apparatus and were free to explore the environment for 10 min. After that, the animals returned to the cage, and one of the objects was replaced by a novel object with a different shape and colour. The apparatus was cleaned with 75% ethanol. After a 2-h interval, mice were allowed to freely explore the area for 5 min, and the time spent exploring the novel object was measured.

### Contextual Fear Conditioning Test

The test was performed as previously reported (Gao et al., 2011). Briefly, on the day of training or test, mice were placed in the conditioning room at least 2 h before fear conditioning. Subsequently, the animals were transferred to the training chamber, freely explored the area for 3 min, received a foot shock (0.7 mA) for 2 s, and returned to the conditioning chamber. Age-matched control animals remained in the conditioning chamber for the same period as the conditioned animals but received no foot shocks. Freezing behaviour was measured for 3 min.

### Stereotaxic Surgery

Experimental mice were anaesthetised using 1.4% isoflurane and placed in a stereotaxic instrument according to a previous report (Hu et al., 2017). The scalp was cut open, and the skull was exposed using 75% ethanol and 1% H_2_O_2_. Double-guide cannulas (Plastics One, USA) were bilaterally implanted into the dorsal hippocampus (anterior-posterior, −1.5 mm; mediolateral, ±1.0 mm, dorsal-ventral, −2.0 mm) using stereotactic manipulators under aseptic conditions. Each cannula contained a dummy cannula and a dust cap (Plastics One, USA) and was fixed to the skull with dental cement. A single injection was performed into each hemisphere using a 33 G Hamilton microinjector in a volume of 0.3 μl and a flow rate of 0.1 μl/min. Needles were gently removed 10 min after injection. The incisions were sutured, and the mice were transferred to a temperature-controlled room (31 ± 1°C).

### Chromatin Immunoprecipitation (ChIP)

ChIP assays were performed using the EZ-Magna ChIP A/G ChIP Kit (Millipore, USA). dCA1 tissues were homogenised in a protease inhibitor solution, fixed in 37% formaldehyde, and cross-linked at room temperature for 30 min. The reaction was inhibited with glycine, and the isolated chromatin samples were sonicated to shear DNA. The cross-linked protein/DNA was immunoprecipitated with a primary antibody at 4°C overnight. The protein/DNA complexes were eluted and reversed cross-links of protein/DNA complexes to free DNA with proteinase K. The purified DNA was collected for PCR analysis (Ding et al., 2017).

### Western Blot Assays

Western blotting was performed as previously reported (Hao et al., 2015). Briefly, dissected dCA1 tissues were homogenised in RIPA buffer containing a protease inhibitor cocktail and a protein phosphatase inhibitor cocktail. The lysate was run on SDS-page gel and transferred to nitrocellulose membranes. The membrane was incubated with the following primary antibodies: anti-BDNF (1:1,000; ab108319; Abcam, UK), anti-H3K9me3 (1:1,000; 13969; Cell Signaling Technology, USA), anti-histone H3 (1:1,000; 4499; Cell Signaling Technology, USA), and β-actin (1:1,000; sc-47778; Santa Cruz Biotechnology, Dallas, TX, USA) at 4°C overnight. The membranes were incubated with horseradish peroxidase-conjugated secondary antibodies (1:1,000; Beyotime Institute of Biotechnology, Haimen, China) and scanned using an infrared imaging system (Bio-Raid, USA).

### Statistical Analysis

Data are presented as the mean ± standard error of the mean. The *Z*-score was calculated based on the results of duration in the novel arm, entries in the novel arm and discrimination index. The formula was *Z*-score = [ΔX_surgery_ − MEAN (Δ_control_)]/SDΔ_control_. All analyses were performed using Prism software. Data normality was assessed using the D’Agostino-Pearson omnibus normality test. Data with a normal distribution were analysed using one-way analysis of variance for multiple comparisons with or without repeated factors, followed by *post hoc* Student–Newman–Keuls (SNK) tests for multiple comparisons. Data without a normal distribution were analysed using non-parametric Kruskal–Willis tests followed by Dunn’s multiple comparisons test. *P*-values for multiple comparisons were adjusted. Data from the two study groups were compared using unpaired Student’s *t*-test. Statistical significance was set at *p* < 0.05.

## Results

### Anaesthesia and Surgery Downregulated BDNF and Impaired Cognition in Middle-Aged Mice

**Figure 1:**
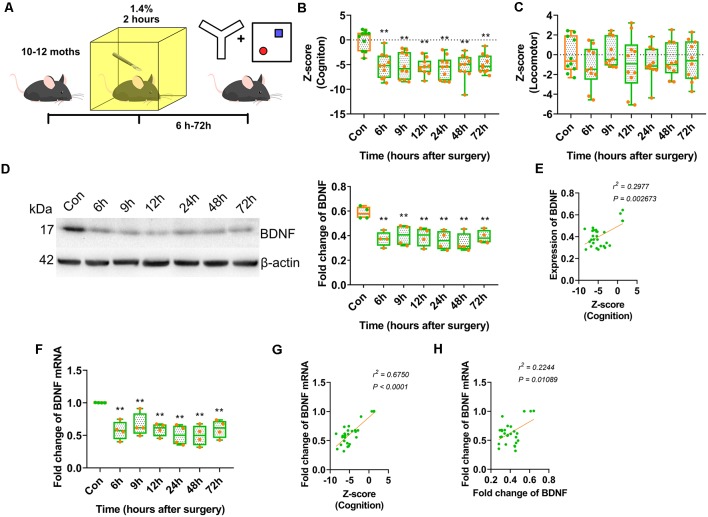
Anaesthesia and surgery induced brain-derived neurotrophic factor (BDNF) repression impaired cognition of middle-aged mice. **(A)** Experimental design. 10–12 month-old C57/BL mice were conduct laparotomy in the chamber filled with 1.4% isoflurane for 2 h. Open filed, Y-maze and novel object recognition (NOR) tests were performed at different time points. The behavioural tests data were collected and calculated the *Z*-score. **(B)** Results of *Z* score for discrimination, duration in novel arm and entries in novel arm (*F*_(6,63)_ = 9.136, *p* < 0.0001; 6 h vs. con, *p* < 0.0001; 9 h vs. con, *p* < 0.0001; 12 h vs. con, *p* < 0.0001; 24 h vs. con, *p* < 0.0001; 48 h vs. con, *p* < 0.0001; 72 h vs. con, *p* < 0.0001, *n* = 10). **(C)** Locomotor tests for mice after anaesthesia and surgery were performed. *Z*-score for locomotor (*F*_(6,63)_ = 0.6341, *p* = 0.7024, *n* = 10). **(D)** The expression of BDNF protein was decreased from 6 h after surgery (*F*_(6,21)_ = 5.564, *p* = 0.0014; 6 h vs. con, *p* = 0.0015; 9 h vs. con, *p* = 0.0063; 12 h vs. con, *p* = 0.0036; 24 h vs. con, *p* = 0.0009; 48 h vs. con, *p* = 0.0003; 72 h vs. con, *p* = 0.0035, *n* = 4). **(E)** The BDNF protein expression was positively correlated to *Z*-score (*r*^2^ = 0.2977, *p* = 0.002673). **(F)** Total BDNF mRNA was changed (*F*_(6,21)_ = 6.490, *p* = 0.0005; 6 h vs. con, *p* = 0.0011; 9 h vs. con, *p* = 0.0089; 12 h vs. con, *p* = 0.0016; 24 h vs. con, *p* = 0.0002; 48 h vs. con, *p* = 0.0002; 72 h vs. con, *p* = 0.002, *n* = 4). **(G)** The BDNF protein expression was positively correlated to *Z*-score (*r*^2^ = 0.5750, *p* < 0.0001). **(H)** The BDNF mRNA expression was positively correlated to BDNF protein expression (*r*^2^ = 0.2244, *p* = 0.01089). All results are represented as $\bar{x}$ ± SEM, **p < 0.01.

We assessed whether anaesthesia and surgery could downregulate BDNF in the hippocampal dorsal CA1 area and whether laparotomy under inhalation anaesthesia in middle-aged mice was a well-established postoperative cognitive impairment model. Middle-aged mice aged 10–12 months underwent laparotomy under general anaesthesia with isoflurane (Peng et al., 2016). The Y-maze and NOR tests were performed to assess cognitive changes induced by anaesthesia and surgery (Figure 1A). All cognitive behavioural data were collected to calculate *Z*-scores. The total distance travelled in the OFT and Y-maze test, and the total number of entries in all arms in the Y-maze test were collected to determine the effect of abdominal surgery on locomotor activity, and *Z*-scores were calculated to analyse changes in locomotor activity.

The *Z*-scores indicated the occurrence of significant cognitive deterioration in the surgery groups (*p* < 0.0001; Figure 1B). However, there were no significant differences in locomotor activity between the surgery and control groups (Figure 1C). These results suggested that anaesthesia and surgery impaired cognition in middle-aged mice but had no significant effect on locomotor activity.

A clinical study reported that BDNF protein expression decreased intraoperatively in cognitively impaired patients. We assessed whether BDNF expression contributed to cognitive dysfunction in an animal model. The expression of BDNF in the dorsal CA1 was decreased in the surgery groups from 6 to 72 h after surgery (*p* = 0.0014; Figure 1D, Supplementary Figure S4A) and was positively correlated with cognition *Z*-scores (*r*^2^ = 0.2977, *p* = 0.002673; Figure 1E). BDNF mRNA expression in this hippocampal region also decreased in the surgery groups from 6 to 72 h after surgery (*p* = 0.0005; Figure 1F) and was positively correlated with cognition *Z*-scores (*r*^2^ = 0.5750, *p* < 0.0001; Figure 1G). Furthermore, BDNF mRNA expression was positively correlated with BDNF protein expression (*r*^2^ = 0.2244, *p* = 0.01089; Figure 1H). These results suggested that the decrease in BDNF expression in the dorsal CA1 region affected cognitive impairment induced by anaesthesia and surgery.

### Postoperative BDNF Downregulation in the Dorsal Hippocampus Impaired Memory Formation but Not Recall

Cognitive processes are complex. The present results suggested that anaesthesia and surgery affected cognition; however, which cognitive processes are impaired is unclear. Contextual fear conditioning experiments were conducted to determine whether anaesthesia and surgery inhibited novel cognitive formation or other cognitive processes.

**Figure 2:**
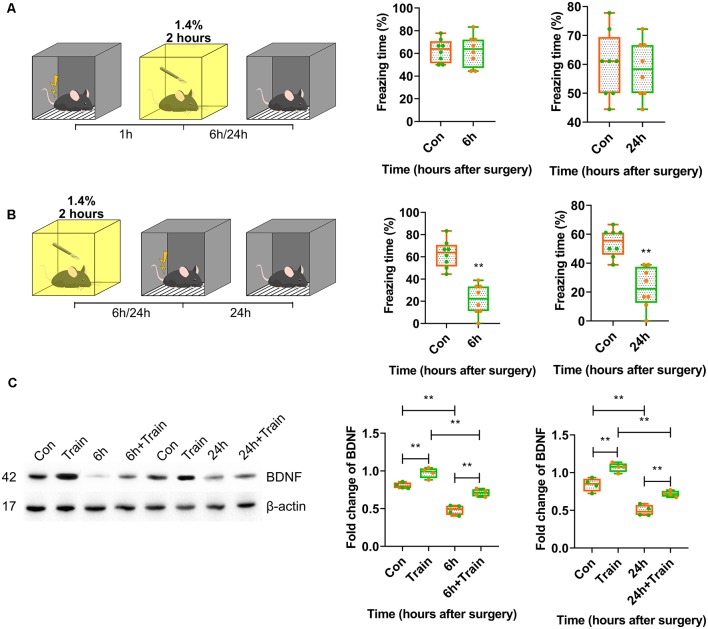
Postoperative BDNF repression in dorsal hippocampus resulted in attenuation of memory acquisition instead of recall. **(A,B)** Contextual fear conditioning behavioural tests for surgery groups. **(A)** Training-surgery-test (6 h vs. Con, *t*_(14)_ = 0.0004114, *p* > 0.9999; 24 h vs. Con, *t*_(14)_ = 0.2607, *p* = 0.7981, *n* = 8) and **(B)** surgery-training-test (6 h vs. Con, *t*_(14)_ = 6.234, *p* < 0.0001; 24 h vs. Con, *t*_(14)_ = 5.167, *p* = 0.0001, *n* = 8) results at 6 and 24 h. Anaesthesia and surgery induced cognition impairment had no effect on formed cognition but inhabited novel cognitive formation. **(C)** BDNF expression changes after training in anaesthesia and surgery group at 6 h (*F*_(3,12)_ = 52.04, *p* < 0.0001; Train vs. Con, *p* = 0.0054; 6 h + Train vs. 6 h, *p* = 0.0005; 6 h + Train vs. Train, *p* = 0.0001; 6 h vs. Con, *p* < 0.0001, *n* = 4) and 24 h (*F*_(3,12)_ = 49.53, *p* < 0.0001; Train vs. Con, *p* = 0.0022; 24 h + Train vs. 24 h, *p* = 0.0027; 24 h + Train vs. Train, *p* < 0.0001; 24 h vs. Con, *p* < 0.0001, *n* = 4). All results are represented as $\bar{x}$ ± SEM, **p < 0.01.

First, training-surgery-test and surgery-training-test experiments were performed to investigate whether anaesthesia and surgery impaired either memory formation or recall. The results showed that anaesthesia and surgery only affected memory formation because fear conditioning was blocked in mice trained after 6 h (*p* < 0.0001) and 24 h (*p* = 0.0001) of surgery, but not before surgery (*p* > 0.9999, *p* = 0.7981; Figures 2A,B). The freezing time in the control group was significantly different than in the iso-train-test group at 6 h (*p* = 0.0441; Supplementary Figures S1A,B).

As previously reported, the BDNF protein expression changes after training represent the fear memory formation. BDNF protein expression changes triggered by foot shock 30 min after training were determined. BDNF expression decreased in mice trained after 6 h and 24 h of surgery (*p* < 0.0001; Figure 2C, Supplementary Figure S4B). In the control group, isoflurane inhalation attenuated BDNF expression triggered by training at 6 h after surgery (*p* < 0.0001; Supplementary Figures S1C, S4E). These results suggested that anaesthesia and surgery inhibited BDNF expression and impaired memory formation but not recall.

### Reversion of the Postoperative Repression of BDNF Expression in the Dorsal Hippocampus Prevented the Impairment of Memory Formation Caused by Anaesthesia and Surgery

**Figure 3:**
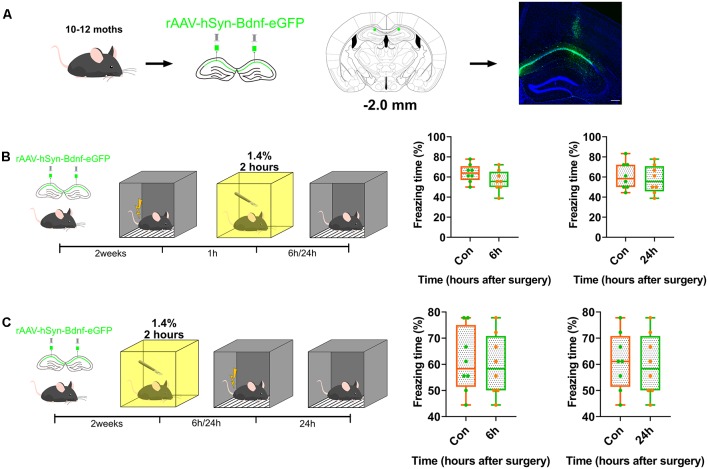
Overexpression of BDNF in dorsal hippocampus rescued the memory impaired by anaesthesia and surgery. **(A)** Diagram of BDNF overexpression in dCA1 of hippocampus and BDNF fused with eGFP infused in neurons. Scale bar, 200 μm. **(B,C)** Over-expression in dCA1 enhanced novel cognition formation both pre- (6 h vs. Con, *t*_(14)_ = 1.571, *p* = 0.1384; 24 h vs. Con, *t*_(14)_ = 0.5047, *p* = 0.6216, *n* = 8) and post-surgery (6 h vs. Con, *t*_(14)_ = 0.2312, *p* = 0.8205; 24 h vs. Con, *t*_(14)_ = 0.2425, *p* = 0.8119, *n* = 8). All results are represented as $\bar{x}$ ± SEM.

BDNF was overexpressed in the dorsal CA1 to confirm whether BDNF downregulation impaired memory formation, and to determine the role of BDNF in contextual fear memory (Figure 3A, Supplementary Table S1). The genetically modified adeno-associated virus overexpressing BDNF was infused into the dorsal CA1 region 2 weeks before fear conditioning tests. The results showed that BDNF overexpression enhanced fear memory at 6 h (*p* = 0.8205) and 24 h (*p* = 0.8119) after surgery (Figures 3B,C) and the vector did not affect the expression of BDNF (Supplementary Figures S3A, S4G).

**Figure 4:**
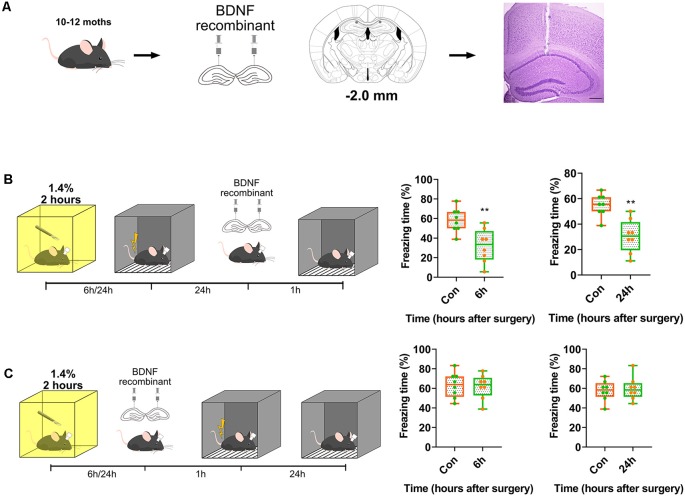
Transient supplement of exogenous BDNF reversed the acquisition of memory instead of recall. **(A)** Diagram of BDNF recombinant was infused into dCA1 of hippocampus. Nissl stain for track of canula. Scale bar, 200 μm. **(B,C)** BDNF into dCA1 enhanced cognition formation (6 h vs. Con, *t*_(14)_ = 3.563, *p* = 0.0031; 24 h vs. Con, *t*_(14)_ = 4.42, *p* = 0.0006, *n* = 8) after surgery rather than recall (6 h vs. Con, *t*_(14)_ = 0.2193, *p* = 0.8296; 24 h vs. Con, *t*_(14)_ = 0.3771, *p* = 0.77117, *n* = 8). All results are represented as $\bar{x}$ ± SEM, **p < 0.01.

Subsequently, we assessed whether exogenous BDNF could enhance memory formation, but not memory recall. Recombinant BDNF was infused into the dorsal CA1 to test whether memory recall was impaired (Figure 4A). The infusion of recombinant BDNF after surgery and training and before the test did not enhance fear memory recall (*p* = 0.0003, *p* = 0.0005; Figure 4B), whereas the infusion of BDNF after surgery and before training significantly enhanced fear memory formation (*p* = 0.8296, *p* = 0.77117; Figure 4C). These results demonstrate that BDNF is involved in memory formation but not memory recall.

### H3K9 Trimethylation Caused the Long-Term Downregulation of BDNF in the Dorsal Hippocampus and Impaired Memory Formation

**Figure 5:**
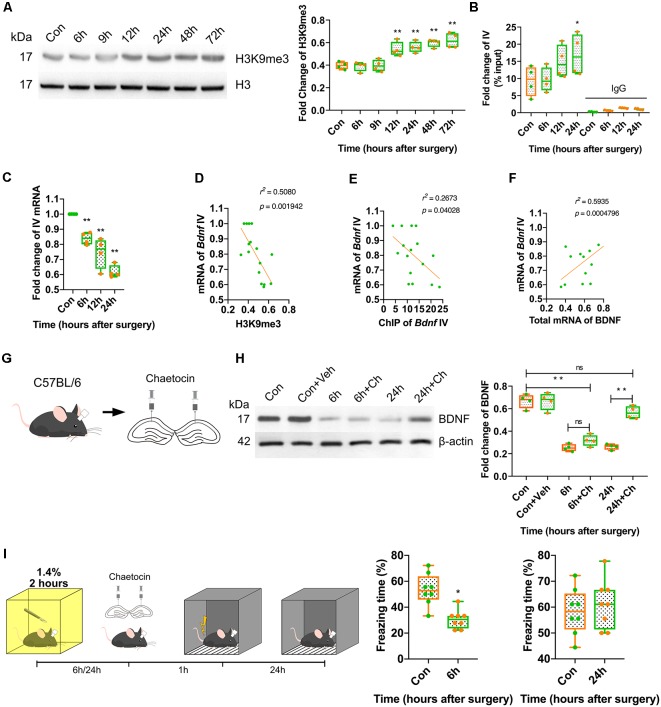
Postoperative trimethylation of H3K9 resulted in long-term BDNF repression in dorsal hippocampus and attenuation of memory acquisition. **(A)** Western blot showed histone H3 occurred modification after surgery and anaesthesia. Results showed H3K9 triple methylation increased from 12 h to 72 h (*F*_(6,21)_ = 16.54, *p* < 0.0001; 12 h vs. Con, *p* = 0.0029, 24 h vs. Con, *p* = 0.0008; 48 h vs. Con, *p* < 0.0001; 72 h vs. Con, *p* < 0.0001, *n* = 4). **(B)** Chromatin immunoprecipitation (ChIP) results showed H3K9me3 clustered with exon IV (*F*_(7,24)_ = 16.79, *p* < 0.0001; 24 h vs. Con, *p* = 0.0318, *n* = 4) was increased at 24 h. **(C)** The expression of the mRNA coded by *Bdnf* exon IV was decreased (*F*_(3,12)_ = 2.548, *p* < 0.0001; 6 h vs. Con, *p* = 0.0056; 12 h vs. Con, *p* = 0.0001; 24 h vs. Con, *p* < 0.0001, *n* = 4). **(D)** The mRNA coded by exon IV of *Bdnf* negatively correlated to the H3K9me3 expression (*r*^2^ = 0.5080, *p* = 0.001942) and **(E)** exon IV ChIP result (*r*^2^ = 0.2673, *p* = 0.04028), **(F)** positively correlated to the total mRNA of BDNF (*r*^2^ = 0.5935, *p* = 0.0004796).** (G)** Diagram of Chaetocin infusion *via* canula fixed in skull stretching to dCA1. **(H)** Western blot results showed Chaetocin abolished BDNF decrease caused by surgery at 24 h, but no effect on 6 h (*F*_(5,18)_ = 56.44, *p* < 0.0001; 6 h + Ch vs. Con, *p* < 0.0001; 24 h + Ch vs. 24 h, *p* < 0.0001; 24 h + Ch vs. Con, *p* < 0.0001; 6 h + Ch vs. 6 h, *p* < 0.0001, *n* = 4). **(I)** Chaetocin reversed the fear memory only at 24 h but not at 6 h after anaesthesia and surgery (6 h vs. Con, *t*_(14)_ = 4.558, *p* = 0.0004; 24 h vs. Con, *t*_(14)_ = 0.607, *p* = 0.5536, *n* = 8). All results are represented as $\bar{x}$ ± SEM; ns, no significance; *p < 0.05, **p < 0.01.

Considering that BDNF expression was repressed from 6 h to 72 h after anaesthesia and surgery, we investigated the mechanism underlying this long-term downregulation. It is known that histone modification plays essential roles in the long-term regulation of transcription, and H3K9 trimethylation mediates transcriptional silencing. The expression of H3K9me3 was measured in this study. The results indicated that H3K9me3 expression increased significantly in the surgery group at 12 h after surgery (*p* < 0.0001; Figure 5A, Supplementary Figure S4C). In the inhalation-only groups, the alteration of H3K9me3 was not observed at all time points (*p* = 0.9085; Supplementary Figures S3B, S4H). Next, we assessed the binding of H3K9me3 to the *Bdnf* exon IV promoter, which is widely distributed in the central nervous system and plays a crucial role in cognition and memory. At 24 h after anaesthesia and surgery, the binding of H3K9me3 to the *Bdnf* exon IV promoter was significantly increased (*p* < 0.0001; Figure 5B), and the expression of the mRNA coded by *Bdnf* exon IV was decreased (*p* < 0.0001; Figure 5C); moreover, the mRNA coded by *Bdnf* exon IV expression was negatively correlated with H3K9me3 expression (*r*^2^ = 0.5080, *p* = 0.001942; Figure 5D) and ChIP results (*r*^2^ = 0.2673, *p* = 0.04028; Figure 5E) and positively correlated with the overall expression of BDNF mRNA (*r*^2^ = 0.5935, *p* = 0.0004796; Figure 5F), suggesting that H3K9 trimethylation was crucial to the long-term downregulation of BDNF.

We hypothesised that BDNF downregulation might be divided into two stages: short-term (up to 24 h) and long-term (more than 24 h). Considering that inhalation anaesthetics are reported to play a role in the rapid inhibition of neuronal activity and that this effect can be prolonged after anaesthesia, we hypothesised that the repression of BDNF expression was induced by isoflurane in the short-term in our study. Therefore, the expression of BDNF was measured in control mice, which received inhalation anaesthesia for 2 h but did not undergo surgery. The results indicated that both the protein expression (*p* = 0.0078; Supplementary Figures S2A, S4F) and total mRNA expression (*p* = 0.0289) of BDNF (Supplementary Figure S2B) were decreased at 6 h after anaesthesia. These findings suggested that the short-term downregulation of BDNF occurred by inhibiting neuronal activity after anaesthesia, whereas the long-term downregulation was in different pathway.

To further determine the effect of H3K9 trimethylation on BDNF expression, histone methyltransferase SUV39H antagonist chaetocin (Ch) was injected into the dorsal CA1 region, which is involved in H3K9 trimethylation (Figure 5G). The inhibition of H3K9 trimethylation restored BDNF expression and reversed the behavioural changes induced by surgery at 24 h but not at 6 h after surgery (*p* < 0.0001; Figures 5H,I, Supplementary Figure S4D) and the vehicle did not affect the BDNF expression (Supplementary Figures S3C, S4I). These results suggested that the long-term downregulation of BDNF triggered by H3K9 trimethylation might be the cause of cognitive and memory impairment postoperatively.

## Discussion

In this study, we demonstrated that anaesthesia and surgery impaired cognition by repressing the expression of the *Bdnf* gene in the dorsal hippocampus, resulting in impairment of memory formation. General anaesthetics can downregulate BDNF expression in the short-term (within 12 h) by inhibiting neuronal activity, resulting in the short-term impairment of memory formation. Anaesthesia and surgery-induced H3K9 trimethylation and the long-term transcriptional repression of BDNF, resulting in long-term (more than 24 h) memory impairment. Given that PND is considered a long-term cognitive impairment, BDNF downregulation induced by H3K9 trimethylation might be the main cause of impairment of memory formation after anaesthesia and surgery.

BDNF is essential for cognition and memory (Bekinschtein et al., 2014) and it is able to cross the Brain-Blood Barrier (BBB) freely for its molecular weight (Klein et al., 2011). BDNF binds to receptor TrkB and activates the phospholipase C (PLC), PI3K, and MAPK/ERK pathways (Minichiello et al., 2002; Yoshii and Constantine-Paton, 2007). Furthermore, BDNF enhances the activation of CREB *via* the MAPK/ERK pathway (Sen et al., 2017). Arc expression is also dependent on this pathway, which is highly related to neuronal activation (Lalonde et al., 2017). Given that memory formation involves the hyperactivity of pyramidal neurons, the downstream activation of BDNF and excitability of pyramidal neurons play a crucial role in cognition and memory. BDNF is known to activate GluA1, a subunit of AMPA receptors, and enhances GluA1 trafficking to the cell surface. BDNF bound to TrkB activates the PLC pathway to enhance calcium signals, consequently activating CaMKII and resulting in GluN2B phosphorylation. Both GluA1 and GluN2B are necessary for long-term potentiation (LTP) and affect cognition and memory. The mechanisms of postoperative cognitive changes are complicated, and it is involved in variety of effects. Multiple reasons such as low temperature, ischemia, stress, inappropriate surgery and anaesthesia might induce the postoperative cognitive changes (Monk et al., 2008; Salazar et al., 2011; Rundshagen, 2014; Robinson et al., 2015; Tian et al., 2015). Almost all the reasons lead to the alteration of BDNF expression after anaesthesia and surgery. In this study, the repression of BDNF expression impaired memory formation directly, and this finding agrees with previous studies.

Epigenetics is reported to be involved in the long-term regulation of cognition and memory without DNA sequence changes. Histone modification is an essential epigenetic mechanism and regulates transcription in cognitive processes (Ding et al., 2017; Kim and Kaang, 2017). It is reported that several sites on histones H2 to H4 are modified by acetylation, methylation, phosphorylation, ubiquitination, and citrullination. Histone H3 methylation is a common way to modify regulation of transcription. H3 methylation results in chromatin condensation, which can inhibit transcription factors that are recruited to DNA binding sites to induce gene silencing. The pharmacological inhibition of H3K9 trimethylation increases BDNF expression in the hippocampus of aged mice (Snigdha et al., 2016). Furthermore, some sites modified in H3, such as H3K9, are located near promotors and cooperate with DNA methyltransferases (DNMTs) to co-repress DNA transcription. H3K9 methyltransferase SUV39H and G9a are also recruited in this complex with DNMT3a or DNMT3b (Fuks et al., 2003; Rai et al., 2010). The deacetylation of H3K9 and H3K14 is regulated by HDAC2 (Wagner et al., 2014; Singh and Thakur, 2018; Watts et al., 2018), recruited and activated by MeCP2 (Mahgoub et al., 2016). The crosstalk between DNA methylation and histone modification allows the stable silencing of the promoters. In our model, some factors promoted the downregulation of BDNF, leading to long-term cognitive impairment after anaesthesia and surgery.

Memory processes are complex, and memory formation is essential for cognitive functioning. Multiple factors are involved in memory formation, and BDNF is reported to play a key role in it. Theoretically, BDNF enhances memory formation but impairs memory retention. *Bdnf* transcription depends on multiple factors, and H3 methylation is directly and indirectly involved in this process. H3K9 trimethylation promotes chromosome condensation and represses BDNF expression, and histone methyltransferase can recruit DNMTs to CpG islands located near the *Bdnf* exons promotors and co-silence *Bdnf* transcription indirectly. These factors impair memory formation and retention, consequently inhibiting LTP.

General anaesthetics target primarily the GABA_A_ receptor (Jurd et al., 2003; Nishikawa and Harrison, 2003; Topf et al., 2003; Winegar and MacIver, 2006; Jia et al., 2008; Ying et al., 2009; Li et al., 2015) and lead to the rapid inhibition of neuronal activity and, ultimately, sedation. BDNF expression was repressed in this study but was short-term, demonstrating that the inhibition of neuronal activity was induced by the activation of GABA_A_ receptors. The prolonged repression of BDNF expression had a significant impact on long-term cognitive impairment induced by anaesthesia and surgery, and this process was highly related to H3K9 trimethylation.

Neuroinflammation is a major contributing factor to cognitive dysfunction. H3K9 trimethylation is reported to be involved in IL-6 regulation (Li Z. et al., 2016), and IL-6 expression is increased in PND patients (Androsova et al., 2015; Li et al., 2017). Furthermore, the disruption of the BBB after surgery may allow more inflammatory molecules to cross this barrier (Li M.-F. et al., 2016). These inflammatory molecules may trigger changes in histone H3 and result in BDNF transcriptional repression, leading to long-term memory impairment after anaesthesia and surgery. Anaesthesia and surgery and postoperative stress may also induce glucocorticoid receptor phosphorylation (Tian et al., 2015), which contributes to postoperative cognitive impairment. Furthermore, H3K9 trimethylation has been reported to be involved in the regulation of stress (Hunter et al., 2012). Despite these known effects, additional studies are needed to elucidate the role of these factors.

In conclusion, the results indicated that BDNF downregulation induced by H3K9 trimethylation impaired memory formation but not memory recall during anaesthesia and surgery. These findings may help understand the molecular mechanisms for PND, and BDNF may be used to prevent postoperative cognitive impairment in the clinic.

## Data Availability Statement

The raw data supporting the conclusions of this manuscript will be made available by the authors, without undue reservation, to any qualified researcher.

## Ethics Statement

The animal study was reviewed and approved by Animal Welfare Committee of Xuzhou Medical University. Written informed consent was obtained from the owners for the participation of their animals in this study.

## Author Contributions

TW and CG conceived and designed the study. TW, X-YS, and KT performed the research. XY, LL, J-RH, YG, and JC contributed data and research tools. TW prepared the manuscript.

## Conflict of Interest

The authors declare that the research was conducted in the absence of any commercial or financial relationships that could be construed as a potential conflict of interest.

## Supplementary Material

FIGURE S1 Inhalation of isoflurane was unable to attenuate the cognition and BDNF expression in dCA1 of hippocampus aroused by training. **(A,B)** Contextual fear conditioning behavioral tests for inhalation-only groups. **(A)** Training-inhalation-test results at 6 (6 h vs. Con, *t*_(14)_ = 0.562, *p* = 0.583, *n* = 8) and 24 h (24 h vs. Con, *t*_(14)_ = 0.3374, *p* = 0.7408, *n* = 8) after inhalation anesthesia. **(B)** Inhalation-training-test results at 6 (6 h vs. Con, *t*_14_ = 2.212, *p* = 0.0441, *n* = 8) and 24 h (24 h vs. Con, *t*_(14)_ = 0.7316, *p* = 0.4765, *n* = 8) after inhalation anesthesia. **(C)** BDNF expression changes after training in anesthesia-only groups at 6 h (*F*_(3, 12)_ = 24.25, *p* < 0.0001; Train vs. Con, *p* = 0.0052; 6 h + Train vs. 6 h, *p* = 0.0008; 6 h + Train vs. Train, *p* = 0.0465; 6 h vs. Con, *p* = 0.0066, *n* = 4) and 24 h (*F*_(3,12)_ = 33.39, *p* < 0.0001; Train vs. Con, *p* < 0.0001; 24 h + Train vs. 24 h, *p* = 0.0001; 24 h + Train vs. Train, *p* = 0.6215; 24 h vs. Con, *p* = 0.9931, *n* = 4). All results were represented as $\bar{x}$ ± s.e.m; ns, no significance; **p* < 0.05, ***p* < 0.01.

FIGURE S2 Inhalation of isoflurane repressed BDNF in dCA1 of hippocampus in short-term. **(A)** Inhalation anesthesia without surgery mice showed changes of BDNF protein at 6 h (*F*_(6,21)_ = 4.019, *p* = 0.0078; 6 h vs. Con, *p* = 0.0429, *n* = 4). **(B)** Total BDNF mRNA was changed at 6 h (*F*_(6,21)_ = 2.980, *p* = 0.0289; 6 h vs. Con, *p* = 0.005, *n* = 4). All results were represented as $\bar{x}$ ± s.e.m; **p* < 0.05, ***p* < 0.01.

FIGURE S3 Inhalation of isoflurane, vehicles and vectors did not affect the H3K9me3 and BDNF expressions. **(A)** The vehicles (*F*_(5,18)_ = 13.74, *p* < 0.0001; Con vs. Con + Veh, *p* = 0.9892; 6 h vs. 6 h + Veh, *p* = 0.9992; 24 h vs. 24 h + Veh, *p* > 0.9999, *n* = 4) and **(B)** vectors (F_(5,18)_ = 16.98 *p* < 0.0001; Con vs. Con + Veh, *p* = 0.9977; 6 h vs. 6 h + Veh, *p* = 0.9999; 24 h vs. 24 h + Veh, *p* = 0.9685, *n* = 4) did not alter the BDNF expression. **(C)**Inhalation anesthesia without surgery mice did not show significant changes of H3K9me3 at all time points (*F*_(6,21)_ = 0.3387, *p* = 0.9085). All results were represented as $\bar{x}$ ± s.e.m; ns, no significance.

FIGURE S4 Full-length pictures of the blots and gels presented in Figure 1 to Figure 5 and Supplementary Figure S1 to Supplementary Figure S3. **(A)** Full-length for Figure 1D. **(B)** Full-length for Figure 2C. **(C)** Full-length for Figure 5A. **(D)** Full-length for Figure 5H. **(E)** Full-length for Supplementary Figure S1C. **(F)** Full-length for Supplementary Figure S2A. **(G)** Full-length for Supplementary Figure S3A. **(H)** Full-length for Supplementary Figure S3B. **(I)** Full-length for Supplementary Figure S3C.

TABLE S1 rAAV-Bdnf titer test report.
