# Supplementary figures and images for: Histone H3K9 Trimethylation Downregulates the Expression of Brain-Derived Neurotrophic Factor in the Dorsal Hippocampus and Impairs Memory Formation During Anaesthesia and Surgery
Source: Front Mol Neurosci. 2019 Oct 25;12:246. doi: 10.3389/fnmol.2019.00246 (PMC6823536; doi:10.3389/fnmol.2019.00246)

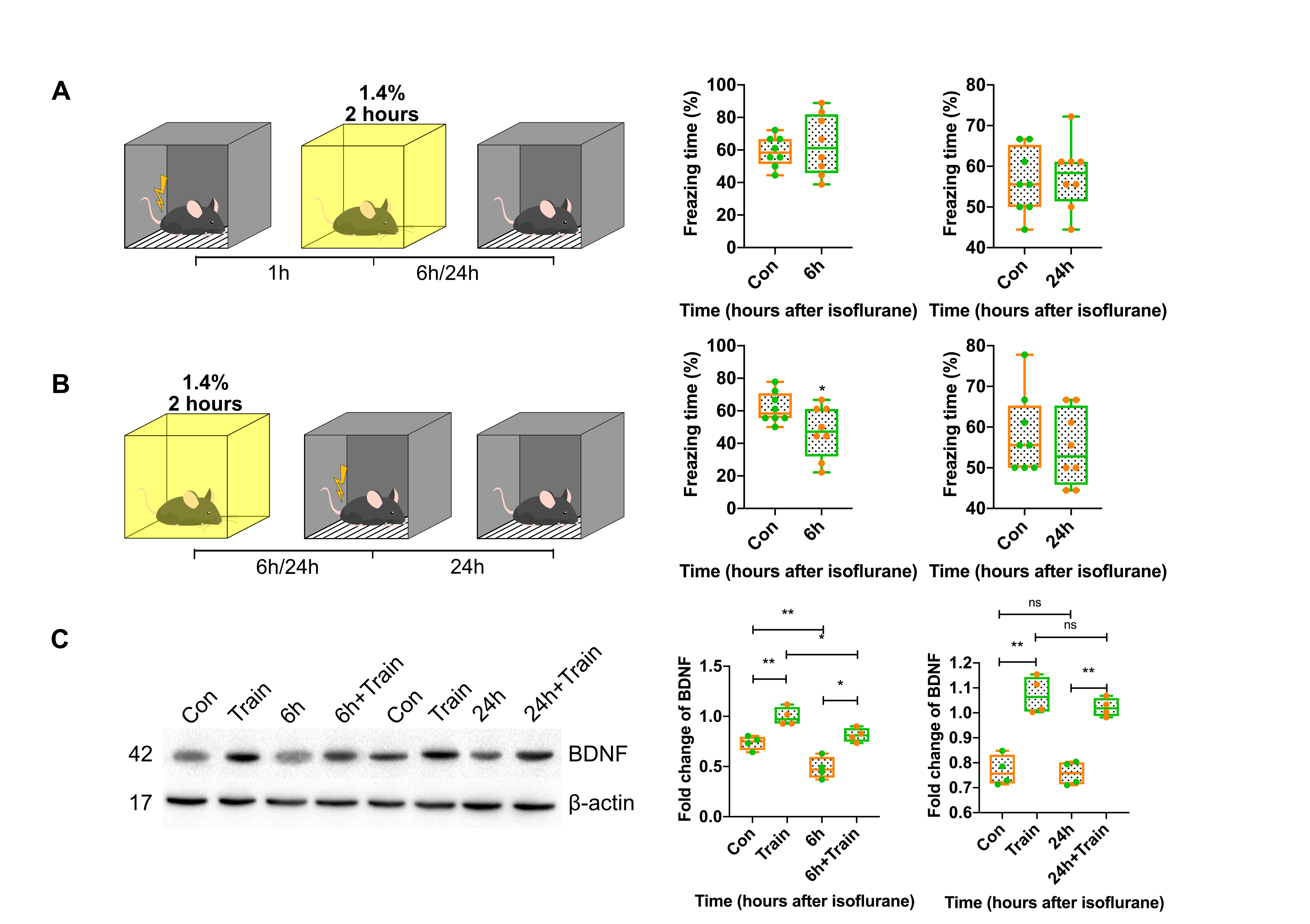

Supplement: FIGURE S1 — Inhalation of isoflurane was unable to attenuate the cognition and BDNF expression in dCA1 of hippocampus aroused by training. (A,B) Contextual fear conditioning behavioral tests for inhalation-only groups. (A) Training-inhalation-test results at 6 (6 h vs. Con, t(14) = 0.562, p = 0.583, n = 8) and 24 h (24 h vs. Con, t(14) = 0.3374, p = 0.7408, n = 8) after inhalation anesthesia. (B) Inhalation-training-test results at 6 (6 h vs. Con, t14 = 2.212, p = 0.0441, n = 8) and 24 h (24 h vs. Con, t(14) = 0.7316, p = 0.4765, n = 8) after inhalation anesthesia. (C) BDNF expression changes after training in anesthesia-only groups at 6 h (F(3, 12) = 24.25, p < 0.0001; Train vs. Con, p = 0.0052; 6 h + Train vs. 6 h, p = 0.0008; 6 h + Train vs. Train, p = 0.0465; 6 h vs. Con, p = 0.0066, n = 4) and 24 h (F(3,12) = 33.39, p < 0.0001; Train vs. Con, p < 0.0001; 24 h + Train vs. 24 h, p = 0.0001; 24 h + Train vs. Train, p = 0.6215; 24 h vs. Con, p = 0.9931, n = 4). All results were represented as x¯ ± s.e.m; ns, no significance; *p < 0.05, **p < 0.01. [file Image_1.TIFF]

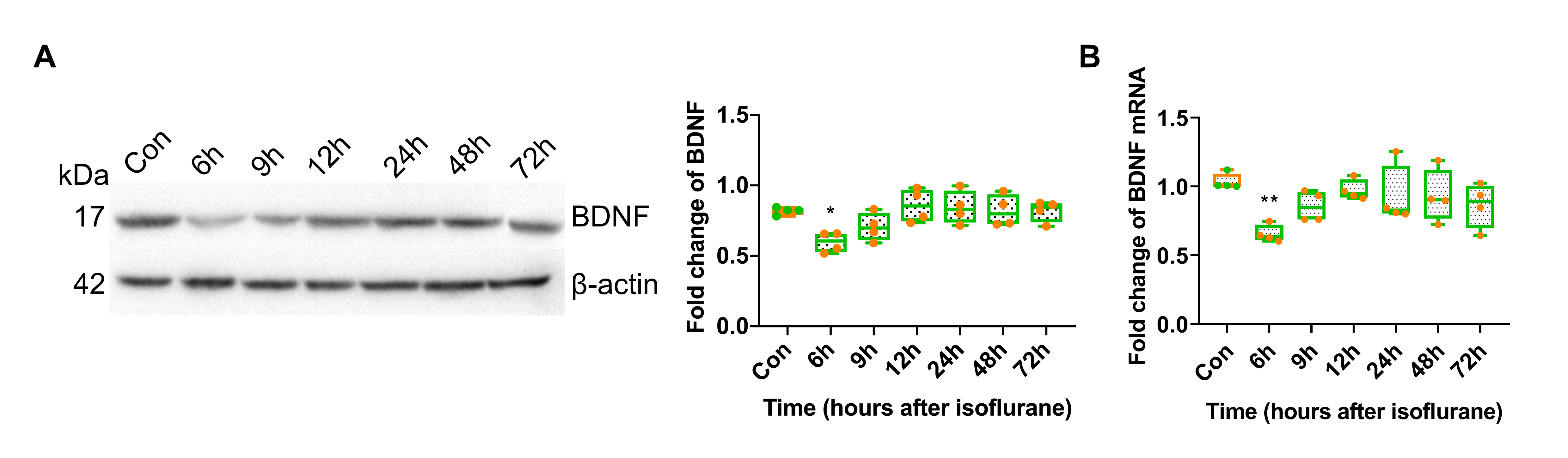

Supplement: FIGURE S2 — Inhalation of isoflurane repressed BDNF in dCA1 of hippocampus in short-term. (A) Inhalation anesthesia without surgery mice showed changes of BDNF protein at 6 h (F(6,21) = 4.019, p = 0.0078; 6 h vs. Con, p = 0.0429, n = 4). (B) Total BDNF mRNA was changed at 6 h (F(6,21) = 2.980, p = 0.0289; 6 h vs. Con, p = 0.005, n = 4). All results were represented as x¯ ± s.e.m; *p < 0.05, **p < 0.01. [file Image_2.TIFF]

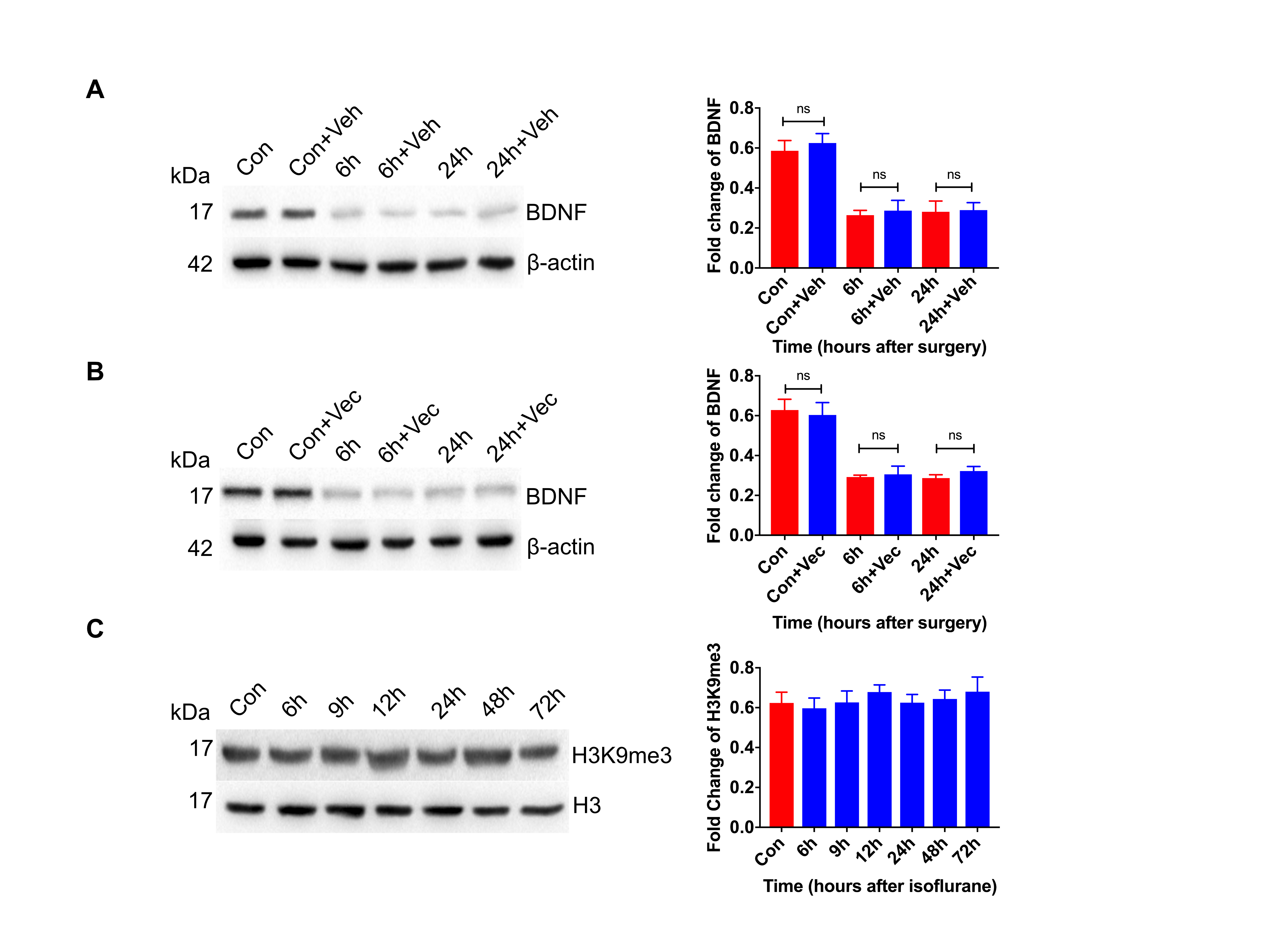

Supplement: FIGURE S3 — Inhalation of isoflurane, vehicles and vectors did not affect the H3K9me3 and BDNF expressions. (A) The vehicles (F(5,18) = 13.74, p < 0.0001; Con vs. Con + Veh, p = 0.9892; 6 h vs. 6 h + Veh, p = 0.9992; 24 h vs. 24 h + Veh, p > 0.9999, n = 4) and (B) vectors (F(5,18) = 16.98 p < 0.0001; Con vs. Con + Veh, p = 0.9977; 6 h vs. 6 h + Veh, p = 0.9999; 24 h vs. 24 h + Veh, p = 0.9685, n = 4) did not alter the BDNF expression. (C)Inhalation anesthesia without surgery mice did not show significant changes of H3K9me3 at all time points (F(6,21) = 0.3387, p = 0.9085). All results were represented as x¯ ± s.e.m; ns, no significance. [file Image_3.TIFF]

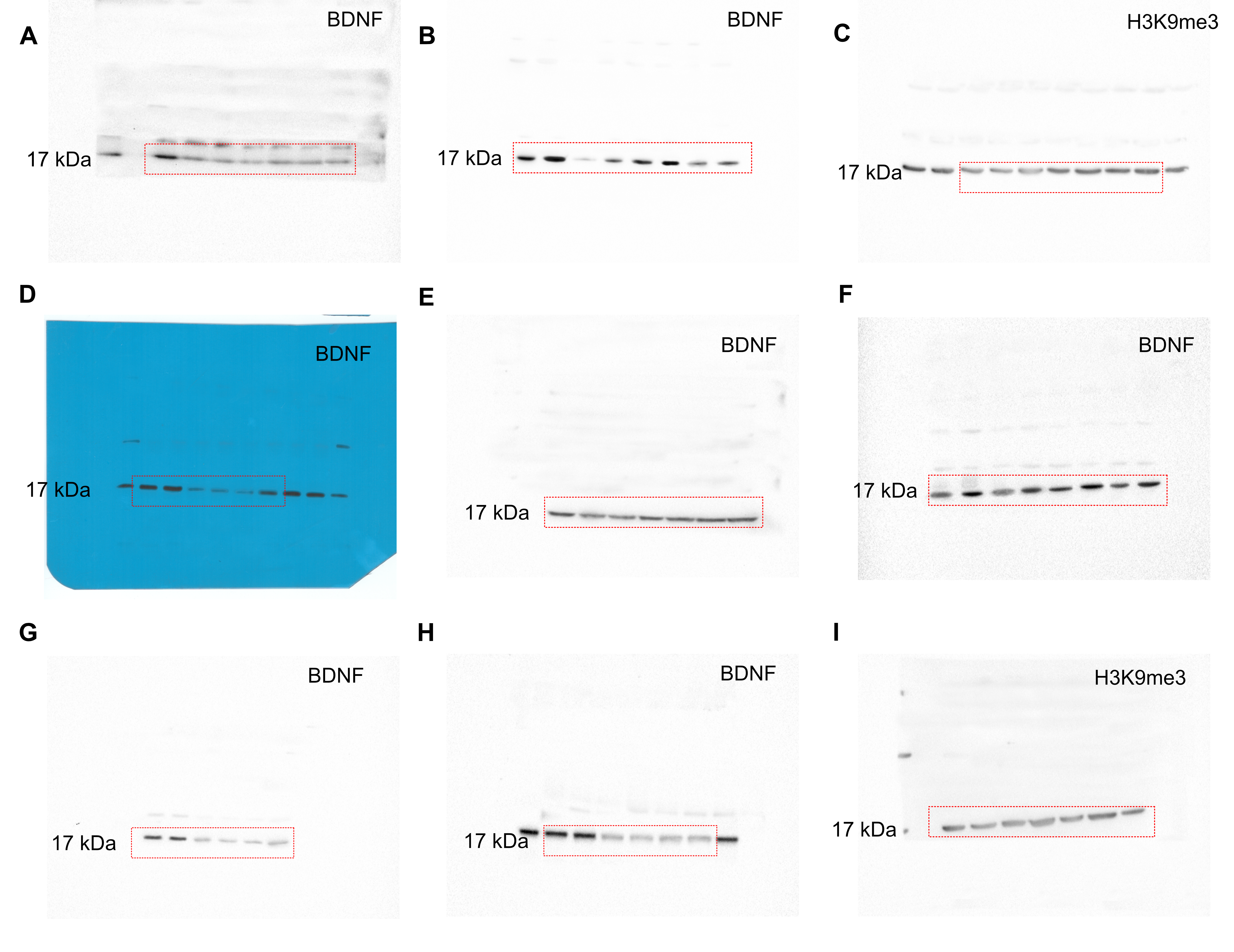

Supplement: FIGURE S4 — Full-length pictures of the blots and gels presented in Figure 1 to Figure 5 and Supplementary Figure S1 to Supplementary Figure S3. (A) Full-length for Figure 1D. (B) Full-length for Figure 2C. (C) Full-length for Figure 5A. (D) Full-length for Figure 5H. (E) Full-length for Supplementary Figure S1C. (F) Full-length for Supplementary Figure S2A. (G) Full-length for Supplementary Figure S3A. (H) Full-length for Supplementary Figure S3B. (I) Full-length for Supplementary Figure S3C. [file Image_4.TIFF]
